# Light incubation affects embryo development and post-hatch performance in poultry

**DOI:** 10.1016/j.psj.2025.105709

**Published:** 2025-08-20

**Authors:** S.Z. Yu, L.F. Cheng, M.Y. He, J. Cao, A.L. Geng

**Affiliations:** a Institute of Animal Husbandry and Veterinary Medicine, Beijing Academy of Agriculture and Forestry Sciences, Beijing 100097, China; b College of Animal Science and Technology, Hebei Agricultural University, Baoding Hebei 071001, China

**Keywords:** Light incubation, Embryo development, Melanopsin, Melatonin, Performance

## Abstract

Light is one of the important environmental factors affecting the growth, reproduction, and performance of poultry. It stimulates retinal photoreceptor cells to form visual imaging, enabling animals to adapt to external environments. Light also regulates non-visual physiological responses such as circadian rhythms, pupillary reflex, sleep regulation, and melatonin secretion. During the incubation, the photoreceptive functions of avian embryo have developed, and light stimulation at this stage affects embryo development and post-hatch performance. This paper mainly reviews the effects of light-emitting diode (LED) light exposure during incubation (light incubation) on embryo development, hatching performance, and post-hatch performance, along with the related mechanisms to provide insights for the research and practical application of light in poultry production.

## Introduction

Light is one of the important environmental factors affecting the growth, reproduction, and performance of poultry. It stimulates retinal photoreceptor cells to form visual imaging, enabling animals to adapt to external environments. Light also regulates non-visual physiological responses such as circadian rhythms, pupillary reflex, sleep regulation, and melatonin secretion. As photo-sensitive animals, the behavior and physiological functions of poultry are strongly influenced by light (Nelson et al., 2020). Artificial light exposure can effectively enhance feed intake and egg production of layers (Liu et al., 2024). Variations of light intensity can modulate physiological and endocrine states of poultry, thereby affecting their behavior and stress responses (Mcphee et al., 2024). Different light spectra (light wavelength, or light color) have different effects on poultry, for example, red light (630 nm) and blue light (460 nm) during brooding period improves survival rates in yellow-feathered broilers compared with white light (Li et al., 2015); monochromatic green light (560 nm) and blue light (480 nm) during incubation enhance muscle growth and myofiber development in broilers (Zhang et al., 2012a,b); white light (380-780 m) primarily influences visual development and mitigates pecking behaviors, etc. (Wang, 2021 ; Tang et al., 2023).

Incubation is the beginning of life in poultry and is one of the most critical phases. Under natural conditions, hens periodically leave their nest to eat, drink, or excrete, so eggs may have some chance of being exposed to sunlight (Duncan et al., 1978). Archer and Mench (2014a) reported that hens typically spend an average of 14.3 d in the nest during incubation, with nest departures increasing during the last week. The eyes, retina, optic nerve and other photosensitive systems of the chicken embryos develop during the incubation (Zhang et al., 2016). Avian embryos respond to light as early as 3 d of development (Erwin et al., 1971). The circadian function of the pineal gland of the brain is mature and becomes secretory at embryonic age 17 (E17, Siri et al., 2013). Therefore, providing light during incubation is conducive to the formation of circadian rhythms in chicken embryos and rhythmic secretion of melatonin (Zeman and Gwinner, 1992), ultimately affecting light adaptation, stress, behavior, and physiology of poultry.

However, in actual production, the factor of light has not yet been utilized. Commercial incubators still adopt a dark environment, possibly due to the secondary heat effects caused by previous light sources, such as incandescent lamp (Gold and Kalb, 1976). With the development and advancement of modern lighting technology, such as the wide application of light-emitting diode (LED) light sources, this problem may be effectively resolved. This paper mainly reviews the effects of LED light exposure during incubation (light incubation) on embryo development, hatching performance, post-hatch performance, and the related mechanisms to provide references for the research and practical application of light in poultry production.

## Light incubation affects embryo development

Light incubation promotes embryo development of *Arbor Acre* broilers (Bai et al., 2017). Providing monochromatic green light (560 nm) during incubation increases the birth weight of chicken embryos of 11.19 %-21.41 % (*P*<0.05) (Feng et al., 2017). Monochromatic green light can significantly enhance the growth of embryos and organs in *Lingnan yellow* chickens (Yu et al., 2018). Providing 400 lux of light during incubation can cause asymmetry in the central nervous system (Rogers, 1982). Different light characteristics, such as light spectrum (Table 1), photoperiod (Table 2), light intensity (Table 3) and light exposure age and duration (Table 4) have different effects on embryo development and performance.

**Table 1:** Effect of light spectrum during incubation on embryo development and performance.

References	Poultry	Light spectrum	Light intensity	Photoperiod, light exposure age and duration	Embryo development and performance
Shafey and Al-mohsen, 2002	Hybro	Dark, Green, 560 nm	1340-1730 lux	24L, E5-15	Green light: embryo weight↑,weight gain↑from E11 to E15, dead embryos↓
Wang et al. (2014)	Arbor Acres	Monochromatic red, Green, Blue, Dark			Green: liver index↑ at E21, proliferating cell nuclear antigen (PCNA) ↑
Huth and Archer, 2015	White Leghorn, Cobb 500, Ross 308.	Dark; White	250 lux	12L:12D, E0-21	White:chick quality↑, hatchability ↑no-defect chick↑
Bai et al., 2016	AA broiler	Dark; White, 400-760 nm; Red, 660 nm; Green, 560 nm; Blue, 480 nm	15 lux	24L, E0-21	Green light: hatching weight↑, muscle indexes↑, muscle fibers↑, number and proliferative activity of satellite cells↑, plasma insulin-like growth factor-1 (IGF-1)↑, skeletal muscle IGF-1 mRNA level↑
Zhang et al., 2016	AA broiler	Dark; White; Green, 560nm	30 lux	24L, E0-19	Green light: weight percentage of yolk retention↓on E19 and 1d post-hatch
Archer, 2017	Cobb 500	Dark; White, 7500 k; Red, 630 nm; Green, 520 nm	250 lux	12L:12D, E0-18	White and Red: hatchability↑, fear response↓, composite asymmetry↓, humoral immunity titers↑; White, Red and Green: non-defect chick↑; All light groups: plasma corticosterone↓, plasma serotonin concentrations↑
Dishon et al., 2017	Cobb 500	Dark; monochromatic green, 560 nm	0.1 W/m^2^	15 min light/ 15 min dark, E0-21	Green light: growth hormone (GH)↑, prolactin (PRL)↑, GHRH↑, growth hormone receptor (GHR)↑, IGF-1 mRNA↑
Sabuncuoglu et al., 2018	Japanese quail	Dark; Green, 560 nm; Blue, 480 nm	1400-1650 lux		Green light: early embryonic mortalities↓; Blue light: late embryonic mortalities↓
Tong et al., 2018	Ross 308	Dark; Green, 460-580 nm	4h 100-130 lux, 1h 1200-1400 lux, 7h 100-130 lux	12L:12D, E0-18	Green light: beak length and crown-rump length↑at E10 and E12, third toe length↑at E10, E14 and E17
Drozdova et al., 2019	Ross 308	Blue, 463 nm; White, 448 nm; Red, 632 nm; Green, 517 nm	0.04 W/m^2^	12L:12D, E11-21	All light: rhythm of pineal melatonin at E20; Melatonin level: Red and White > Green > Blue
Wang et al., 2020	White Leghorn, Rhode Island Red, Columbia Rock, and Barred Rock	Dark; Green, 520-525 nm	200 lux	12L:12D, E0-18	Green light: hatch time, 90 % hatch time, and average hatch time↓, Rhode Island Red: green light body weight↓than dark
Li et al., 2021a	Ross 308	Dark; White, 4100 k; Red, 640 nm; Blue, 460 nm	200 lux	12l:12D	Red light: air cell temperature↓
Wang, 2021	White Leghorn, Rhode Island Red, Columbia Rock, and Barred Rock	Dark; Green, 560nm	200 lux	12L:12D, E0-18	Green light: liver weight and pineal development↑
Tang et al., 2023	Jinghong No.1 Layer	Dark; White, 380-780 nm; Blue, 455/447.5-462.5 nm; Green, 525/515-535 nm	4h 200±50 clux, 1h 2000±500 clux, 7h 200±50 clux	12L:12D, E0-21	Hatching time: white↑, green↑; White: retinal thickness↑; Melatonin: green and blue↑
Guo et al., 2024	White King pigeons	Dark; White, 400-760 nm; Red, 660 nm/620-700 nm; Green, 560 nm/535-585nm	100 lux	12L:12D, E0-15	White: hatching time↓than dark; Hatchability: white↑, red↓; Green: embryo weight and relative leg muscle↑on E14 and d 1 compared to dark
Wei et al., 2025	Jingfen No.6 layer	Dark; White, 5000 K; Green, 520/515-525nm	4h 200±50 lux, 1h 2000±500 lux,7h 200±50 lux	12L:12D, E0-21	Retina thickness: white↑than green light at E20; White: TGF-β1 mRNA and Smad 2/3 protein↓; Green light: cBmal1/2, cClock and cAanat diurnal rhythms, melatonin secretion↑

**Note:** 24L: 24 h light; 12L:12D: 12 h light and 12 h darkness; E: embryonic day. E5-15: light exposure from E5 to E15. E0-15: light exposure from E0 to E15. E0-18: light exposure from E0 to E18. E0-19: light exposure from E0 to E19. E0-21: light exposure from E0 to E21. E11-21: light exposure from E11 to E21.

All indicators listed in the table exhibit statistically significant differences (*P*<0.05).

**Table 2:** Effects of photoperiod during incubation on embryo development and performance.

References	Poultry	Photoperiod	Light intensity	Light spectrum	Embryo development and performance
Archer and Mench, 2013	Cobb 500	Inc_24D_, Inc_1L:23D_, Inc_6L:18D_, Inc_12L:12D_	550 lux	White light, 5000 K	Inc_12L:12D_: corticosterone↓after 1 h of crating, composite asymmetry↓on d 1 post-hatch
van der Pol et al., 2019a	Ross 308	Inc_24D_, Inc_12L:12D_, Inc_24L_	500 lux	White light, 6050 K (420-780 nm, peak 454 nm)	Inc_24L_: embryonic ossification↓at E13 and E14, femur length, tibiatarsus weight and length, cortical area, and mean cortical thickness↓at hatching on D0 compared to Inc_12L:12D_; Inc_24D_: growth hormone↑at D0 than Inc_12L:12D_
van der Pol et al., 2019b	Ross 308	Inc_24D_, Inc_16L:8D_, Inc_24L_	500 lux	White light, 6050 K (420-780 nm, peak 454 nm)	Inc_24D_: femur length and femur and tibia weight, width↑at hatch than Inc_16L:8D_, Inc_24D_: femur length and width and tibia depth↑than Inc_24L_; Inc:_16L:8D_: dark period pineal melatonin↑at 458 h of incubation than for Inc_24L_ and Inc_24D_
Geng et al., 2021	Beijing You Chicken	Inc_24D_, Inc_8L:16D_, Inc_12L:12D_, Inc_16L:8D_	150-200 lux	White, 4500-5000 K	Inc_12L:12D_ and Inc_16L:8D_: leg problems↓; Inc_12L:12D_: femur length↑, lysozyme content↑, Glutathione Peroxidase (GSH-Px)↑and Total Superoxide Dismutase (T-SOD) activities↑
Li et al., 2023	Cobb 500 and Ross 308	Inc_24D_, Inc_12L:12D_, Inc_18L:6D_	200 lux	Blue light	Inc_12L:12D_ and Inc_18L:6D_: air cell temperature↓at E13; Cobb embryos: Inc_12L:12D:_ air cell temperature↓than Inc_18L:6D_ at E16; Ross embryos: Inc_18L:6D_ air cell temperature↓than Inc_12L:12D_; Inc_12L:12D_: navel closure↑; Inc_12L:12D_: body weight ↑on d 14 body weight gain↑than Inc_18L:6D_

**Note:** Inc: incubation; Inc_24D_: 24 h dark; Inc_12L:12D_: 12 h light and 12 h dark; Inc_18L:6D_: 18 h light and 6 h dark; Inc_24L_: 24 h light; E: embryonic day.

All indicators listed in the table exhibit statistically significant differences (*P*<0.05).

**Table 3:** Effects of light intensity during incubation on embryo development and performance.

References	Poultry	Light intensity	Light spectrum	Photoperiod	Embryo development and performance
Yu et al., 2018	Broiler	300 lux, 150 lux, 50 lux, Dark	Green,560 nm		50 lux: embryo weight↑, body length↑, hatchability↑, and thyroxine↑
Hao, 2020	Bashang Long Tail Chicken, AA broiler	150-200 lux, 90-140 lux, 30-80 lux, Dark	Green,525 nm	12L:12D, E10-18	30-80 lux: no yolk body weight↑; AA+ broiler: pectoral muscle fiber diameter↑,duodenum, jejunum, and ileum↑; Bashang Long Tail Chicken: duodenal villi and jejunal villi↑; light group: Bashang Long Tail Chicken: pectoral muscle fiber diameter↑and bursa follicle area↑than dark, AA+ broiler the diameter of leg muscle fiber↑; 150-200 lux: the duodenal villi and ileal villi↓

**Note:** 12L:12D: 12 h light and 12 h dark. E: embryonic day. E10-18: light exposure from E10 to E18.

All indicators listed in the table exhibit statistically significant differences (*P*<0.05).

**Table 4:** Effects of light exposure age and duration during incubation on embryo development and performance.

References	Poultry	Light exposure age and duration	Light spectrum	Light intensity	Photoperiod	Embryo development and performance
Özkan et al., 2012a	Ross 308	Dark, E0-21, E14-21	White, 4000 K	200-300 lux	16L:8D	E0-21: embryo weight/egg weight ratio↑at E13 and E18, residual yolk weight↓than E14-21 at E18; E0-21 and E14–21: daily rhythm of melatonin at hatching; E14–21: breast muscle weight ↑
Özkan et al., 2012b	Ross 308	Dark, E0-21, E14-21	White, 4000 K	200-300 lux	16L:8D	E0–21 and E14–21: body weight↑than Dark at 35 d; Light groups: wight gain↑,breast muscle weight↑at E6, blood corticosterone levels↓, malondialdehyde concentration in brain tissue↓
Dishon et al., 2018	Cobb 500	Dark, E0-20, E10-20, E15-20	Green, 560 nm	0.1 W/m^2^	15 min light/ 15 min dark	E0-20: growth hormone (GH)↑, hypothalamic growth hormone releasing hormone (GHRH)↑, and liver growth hormone receptor (GHR)↑, insulin-like growth factor-1 (IGF-1) mRNA levels↑, GH plasma levels↑compared to Dark; E15-20: plasma GH levels↑
Dishon et al., 2021	Cobb 500	Dark, E0-20, E15-20, E16-20, E17-20, E18-20	Green, 560 nm	0.1 W/m^2^	15 min light/ 15 min dark	E18-20, E0-20, similar in body weights↑, breast muscle weights↑, somatotropic axis activity↑

**Note:** Inc: incubation; 16L:8D: 16 h light and 8 h dark; E: embryonic day; E0-21: light exposure from E0 to E21; E14-21: light exposure from E14 to E21; E15-20: light exposure from E15 to E20; E16-20: light exposure from E16 to E20; E17-20: light exposure from E17 to E20; E18-20: light exposure from E18 to E20;

All indicators listed in the table exhibit statistically significant differences (*P*<0.05).

### Light incubation affects pineal gland development

The pineal gland is the main photo-sensitive and endocrine organ of poultry. The pineal gland of poultry is located at the triangular gap between the cerebral hemisphere and cerebellum, with its top connected to the dura mater and its slender stalk connected to the third ventricle of the brain. Light incubation promotes the enlargement of follicular area, wall thickness, and luminal area in the embryonic pineal gland (Wang, 2021). Additionally, light regulates the pineal gland’s development and maturation, thereby influencing melatonin secretion (Tang et al., 2023) and the formation of circadian rhythm (Drozdova et al., 2019).

### Light incubation affects visual development

Light incubation significantly affects the visual organ development of poultry. Tang et al. (2023) investigated the effects of different light spectra (white light, 380-780 nm; green light, 525 nm; blue light, 455 nm) during incubation and found a notable increase in retinal thickness in chicks, with white light exerting the most pronounced effect (Wei et al., 2025). Archer et al. (2009) reported that chicks incubated under 24 h light (Inc_24L_) or 24 h darkness (Inc_24D_) exhibited heavier eye weights compared to those exposed to 12 h light and 12 h darkness (Inc_12L:12D_). Gorla and Nag (2021) observed significant damage to rod and cone ganglion cells in chicks after exposing to white light (700 lux) from E1 to E7 followed by continuous 24 h light (700 lux) from E7 to E18. Li et al. (2023) observed that Inc_16L:8D_ significantly reduced post-hatch feed intake within the first 6 h compared to dark or Inc_12L:12D_ (*P*<0.05), indicating that 16 h light may cause some degree of stress and have negative effects on early performance. These findings highlight the need to optimize light regimes during incubation to ensure favorable impacts on embryo development and subsequent growth in poultry.

### Light incubation affects myofiber development

Most of research on the effects of light incubation on myofiber development have predominantly focused on green light. Studies showed that monochromatic green light (560 nm) during incubation increases the myofiber cross-sectional area by 10.1 % to 103.9 % (*P*<0.05) compared to red (660 nm), blue (480 nm), white (400-700 nm), and darkness (Bai, 2017). Monochromatic green light incubation (560 nm) promotes embryonic myocyte proliferation and breast muscle weight in turkeys (Rozenboim et al., 2003), increases body weight and muscle development in broiler (Dishon et al., 2017; Xie et al., 2009; Zhang et al., 2012), enhances muscle antioxidant capacity (Yu, 2014), and promotes muscle growth in broilers, potentially mediated by activation of the growth axis to stimulate myocyte proliferation, especially myoblast proliferation, creating a favorable environment for myoblast expansion in later incubation stages (Halevy et al., 2006; Halevy, 2020). It also influences the proliferation and differentiation of satellite cells in chicks (Bai, 2017).

Monochromatic green light incubation stimulates the embryonic endocrine system, elevates secretion of growth axis hormones, such as growth hormone (GH) and insulin-like growth factor-1 (IGF-1) (Bai et al., 2016; Zhang et al., 2012), which regulate skeletal muscle satellite cell proliferation via the intracellular PI3K/Akt signaling pathway (Li et al., 2016). Monochromatic green light incubation increases body weight gain in broilers at 21 and 42 d post-hatch, with the highest breast muscle weight and yield observed at 42 d of age compared to dark and blue light groups (Zhang et al., 2012); The reason may be due to that monochromatic green light upregulates mRNA expression of GH receptor and myostatin in breast muscle during late embryogenesis (E17) and growth phases (21 d post-hatch) (Zhang et al., 2014); Moderate-intensity monochromatic green light (525 nm, 90-140 lux) significantly enhanced leg and breast myofiber diameter and leg muscle cross-sectional area in *Bashang Long-tailed* chickens compared to low-intensity (30-80 lux), high-intensity (150-200 lux), and dark groups, though high-intensity light suppressed breast myofiber development (Hao, 2020).

### Light incubation affects leg bone development

Light incubation significantly influenced embryonic leg bone development in poultry (Table 5). Yu (2016) demonstrated that incubation from E0 to E7 (green light, 525 nm, 150-392 lux) significantly promoted tibial length in chicks at hatching compared to those from E0 to E14 and from dark group. Tong et al. (2018) found that monochromatic green light (460-580 nm) significantly increased beak length and crown-rump length at E10 and E12, while the third toe length was significant longer at E10, E14, and E17 compared to the dark group. van der Pol et al. (2017) investigated the effects of three light regimes—continuous 24 h light (Inc_24L_), 16 h light/8 h darkness (Inc_16L:8D_), and continuous 24 h darkness (Inc_24D_) (500 lux), combined with two post-hatch light programs (24L:0D or 16L:8D) on leg bone development in Ross 308 broilers, and revealed that the Inc_24L_ group exhibited more growth plate abnormalities than the Inc_16L:8D_ or Inc_24D_ groups, while the Inc_24D_ group showed a higher incidence of bacterial chondronecrosis compared to Inc_16L:8D_, suggesting that the Inc_16L:8D_ could improve leg health in broilers. The Inc_12L:12D_ was found to enhance long bone growth and reduce leg-related issues in 1-d-old native chicks compared to Inc_24D_ (Geng et al., 2021).

**Table 5:** Effects of photoperiod during incubation on post-hatch performance.

References	Poultry	Photoperiod	Light Intensity	Light spectrum	Post-hatch performance
Archer et al., 2009	Cobb 500	Inc_24D_, Inc_12L:12D_, Inc_24L_	550 lux	White light	Inc_24L_ and Inc_12L:12D_: feeding activity↑; Inc_12L:12D_: body weight↓; Inc_24D_: composite physical asymmetry↑
van der Pol et al., 2017	Ross 308	Inc_24D_, Inc_16L:8D_, Inc_24L_	500 lux	whit light 6050K	Inc_24L_: body weight↑, epiphyseal plate abnormalities↑; Inc_24D_: BCO↑than Inc_16L:8D_. Inc_24L_: femur mineral density↑
Li et al., 2023	Cobb 500 and Ross 308	Inc_24D_, Inc_12L:12D_, Inc_18L:6D_	200 lux	Blue light	Inc_12L:12D_: navel closure↑; Inc_12L:12D_: body weight↑on d 14 body weight gain↑than Inc_18L:6D_

**Note:** Inc: incubation; Inc24D: 24 h dark; Inc12L:12D: 12 h light and 12 h dark; Inc24L: 24 h light.

BCO: Bacterial Chondronecrosis with Osteomyelitis

All indicators listed in the table exhibit statistically significant differences (*P*<0.05).

van der Pol et al. (2019a) investigated three incubation light regimes, Inc_24L_, Inc_12L:12D_ and Inc_24D_, and found that the Inc_12L:12D_ group showed a higher tibial ossification rate between E12 and E14, along with longer tibiae and femora in hatchlings. They suggested that Inc_12L:12D_ improved leg bone development, while Inc_24L_ had a greater adverse effect on embryonic bone development and post-hatch leg bone strength compared to Inc_12L:12D_ and Inc_24D_, confirming the adverse impacts of Inc_24L_ on embryonic bone development, and revealing a higher incidence of tibial chondrodysplasia in the Inc_24L_ group than in Inc_12L:12D_, with these negative effects persisting into the post-hatch (van der Pol et al., 2019b). However, Güz (2021) reported no beneficial effects of green light incubation (Inc_16L:8D_) on post-hatch tibial development, which may be attributed to the specific light spectrum (green light, 522 nm) and photoperiod (Inc_16L:8D_).

### Light incubation affects gonadal development

Light acts on deep-brain photoreceptors within the hypothalamus, converting optical signals into neural impulses that regulate reproductive activities via the hypothalamic-pituitary-gonadal (HPG) axis (Cheng et al., 2021). The hypothalamus secretes gonadotropin-releasing hormone (GnRH) and gonadotropin-inhibitory hormone (GnIH), which modulate the release of pituitary gonadotropins. These hormones circulate in the bloodstream to influence gonadotropin secretion, thereby promoting gonadal development and regulating animal reproduction (Zhou et al., 2022). During incubation, asymmetric light exposure to the eyes can lead to asymmetrical embryonic development (Ciacciariello and Gous, 2010). Red light stimulates gonadal development in poultry, and has a positive effect on sexual maturation, whereas blue and green light suppress gonadal growth but may promote gonadal maturation (Li and Wang, 2021).

### Light incubation affects navel closure

During the early and middle stages of chicken embryo development, the abdomen remains in a semi-open state with the yolk sac and portions of the intestine exposed outside. From E15 to E19, the small intestine gradually retracts into the body cavity, and the body wall gradually closes to form a circular navel, with the yolk sac progressively internalized in later stages. Asynchronous development of the embryo’s small intestine and yolk sac, along with risk factors such as older laying hens, prolonged egg storage, or suboptimal incubation temperatures (either excessively high or low), increases the incidence of poor navel closure in hatchlings. Poor navel closure is an indicator of inefficient nutrient utilization from the yolk sac, which increases the risk of omphalitis and affects later growth. Studies have shown that light incubation impacts navel closure in embryos. For example, Inc_12L:12D_ (200 lux) enhanced navel closure condition of hatchlings compared to dark group (*P*<0.05) (Li, et al., 2023), which was similar to what Huth and Archer (2015) observed that periodic illumination improved naval quality of newly hatched chicks. The improved navel closure may relate to the positive effect of periodic illumination on embryo development, which altered hypothalamus-pituitary-thyroid (HPT) axis activity and improved navel maturation (Li et al., 2023). White and red light (630 nm) increase the proportion of Ross 308 broilers with well-healed navels (Archer, 2017).

## Light incubation affects hatching performance

Hatchability is an important economic indicator in poultry production. Factors influencing hatchability include the fertility rate of breeder eggs, hatching temperature, relative humidity, location of breeder eggs, egg-turning practices, and embryo positioning within the egg. Light incubation also affects hatchability, with varying impacts observed across different light characteristics, such as light spectrum, photoperiod, light intensity and light exposure age and duration.

### Effect of light spectrum on hatchability

Yu et al. (2018) found that monochromatic green light improved the hatchability of *Lingnan Yellow* chickens by approximately 6 % (*P*<0.05). Shafey and Al-mohsen (2002) reported that green light (560 nm) at 1,340–1,730 lux from d 5 to d 15 of incubation accelerated embryo development and increased hatchability by 4.8 % (*P*<0.05). Huth et al. (2015) demonstrated that white light (250 lux) incubation significantly enhanced hatchability and chick quality in broiler breeds (Ross 308 and Cobb) compared to that in dark incubation. Archer (2017) observed that white and red light (630 nm, 250 lux) improved hatchability in Ross 308 broilers by reducing early embryonic mortality in fertilized eggs. Different light spectra significantly increased hatchability of King White Pigeon eggs compared to that in darkness, while red light (660 nm, 100 lux) reduced it (Guo et al., 2024).

Sabuncuoglu et al. (2018) tested monochromatic green light (560 nm) and blue light (480 nm) in Japanese quail, and found no significant differences in overall hatchability or total embryonic mortality. However, the green light group exhibited the lowest early embryo mortality (12.37 %) (*P*<0.05), whereas the blue light group showed the lowest late embryo mortality (13.59 %) (*P*<0.05). Yu (2016) applied green light (525 nm) to *Lingnan Yellow* broiler eggs (light-colored shells) and found that a 22–75 lux green light increased hatchability by approximately 6 % (*P*<0.05). These studies suggest that some light spectra during incubation can reduce embryo mortality, contributing to improved hatchability. Yu et al. (2022) proposed that green light (525 nm, 120-220 lux) incubation enhanced the absorption of calcium by the eggshell, increased the pore size of the eggshell surface during incubation, and improved the permeability and heat dissipation of the eggshell compared to dark group. These changes facilitate chick pipping, thereby promoting hatching success and reducing incubation mortality.

However, some studies report no effect of some light spectrum on hatchability. Tang et al. (2023) proposed that different light spectra (white, 380 nm; blue, 455 nm, and green, 525 nm) had no impact on hatchability compared to dark group. Wang et al. (2020) compared the effects of monochromatic green light (520-525 nm, 200 lux) on four breeds of hatching eggs (White Leghorn, Barred Plymouth Rock, Columbian Plymouth Rock, and Rhode Island Red) and found no differences in hatchability. Güz (2021) observed no influence of green light (522nm, 288lux) on hatchability in Ross 308 breeder eggs. Li et al. (2021) reported no significant changes in hatchability or embryo mortality under red, white, or blue light (455 nm, 200 lux). Huth et al. (2015) noted that white light (250 lux) improved hatchability in pigmented eggs but had no effect on white-shelled eggs (White Leghorn). In summary, the impact of light spectrum on hatchability varies across studies. Most reports suggest improved hatchability under monochromatic green light, while white and red light may benefit pigmented eggs (Archer, 2014a, 2016), and blue light exhibits minimal effects (Li et al., 2023).

### Effect of light intensity and photoperiod on hatchability

Compared to light spectrum, fewer studies have focused on light intensity and photoperiod. Li et al. (2023) found that blue light under different photoperiods (24D, 18L:6D, and 12L:12D) had no significant impact on hatchability but accelerated body weight and weight gain in chicks by d 14 post-hatch. Geng et al. (2021) revealed that varying durations of white light exposure (150-200 lux) during incubation did not affect hatchability, or chick health in *Beijing You Chicken*. Yu et al. (2018) investigated the effects of green light (560 nm, 150-392 lux) on *Lingnan Yellow* chickens and observed that only low-intensity light (50 lux) increased thyroid hormone levels in hatchlings compared to high-intensity light (150 lux and 300 lux), while high-intensity light (150 lux and 300 lux) reduced thyroid hormone and testosterone levels compared to those in the dark group. Shafey and Almohsen (2002) reported that high-intensity green light (fluorescent light, 560 nm, 1,340-1,730 lux) during incubation improved hatchability of meat-type breeder eggs (Hybro).

### Effect of light incubation on hatching time

Teng et al. (2016) reported that white light accelerates poultry embryo development and reduces hatching time compared to dark group. Tong et al. (2018) found that 12L:12D green light (460-480 nm) advanced the hatching time of broiler eggs by 3.4 h compared to the control group, correlating with accelerated secretion of thyroid hormones and corticosterone. Yu (2016) demonstrated that monochromatic green light (525 nm) advanced the peak hatching period by 12 h relative to darkness. Zhong (2018) also observed that green light (120-220 lux) shortened the total hatching time and the peak hatching window, which may be due to the enhanced nutrient utilization, yolk absorption efficiency, and hatching-related hormone secretion. Wang et al. (2020) observed that monochromatic green light (520-525 nm, 200 lux) shortened the average hatching time by 5.34 h (*P*<0.05) for *White Leghorn, Barred Plymouth Rock, Columbian Plymouth Rock*, and *Rhode Island Red* eggs, without extending the hatching window or peak hatching period.

However, some studies reported no effect of light on hatching time. Özkan et al. (2012b) found no significant changes in hatching time under cool light incubation. Tang et al. (2023) incubated *Jinghong No.1* layer eggs under different spectra (12L: 12D; white light, 380-780 nm; blue light, 455 nm; green light, 525 nm) and found that the average incubation time was delayed by 7.5 h (white light) and 5.5 h (green light) compared with the control group (*P*<0.05), respectively. The above differences may be related to the genetic variations in the breeder eggs (Hannah et al., 2020).

## Effects of light incubation on post-hatch performance

Light incubation affected the development of chicken embryos, and these effects persisted into the post-hatch period (see Table 6).

**Table 6:** Effects of light spectrum during incubation on post-hatch performance.

References	Poultry	Light spectrum	Light intensity	Photoperiod	Post-hatch light	Post-hatch performance
Rozenboim et al., 2003	Turkey	White; Green, 560 nm; Dark	0.14 W/m^2^	3 min light:3 min dark		Green light: female turkeys had greater body weight↑than others from 28 d to 79 d, breast muscle weight↑
Zhang et al., 2012	AA Broiler	Green, 565 nm; Blue, 480 nm; Dark	15 lux		White, 30 lux, 23L:1D	Green light: body weight↑, breast muscle weight↑, feed intake↑than others, feed conversion ratio↓than dark
Zhang et al., 2014	AA Broiler	Green, 565 nm; Dark	15 lux		White, 30 lux, 23L:1D	Green light: body weight, pectoral muscle weight and myofiber cross-sectional areas↑than dark on d 7, satellite cell mitotic activity of pectoral muscle↑on d 1 and d 3, MyoD, myogenin and myostatin mRNA expression↑in newly hatched chicks.
Huth and Archer, 2015	White Leghorn; Cobb 500, Ross 308.	Dark; White	250 lux	12L:12D, E0-21		Light: physical asymmetry↓, heterophil/lymphocyte ratios↓at 14 d grow-out
Archer et al., 2017	Cobb 500	White, 7500k; Red, 630 nm; Green, 520 nm; Dark	250 lux	12L:12D	20L:4D, 20-50 lux	White and Red: hatchability↑, fear response↓, composite asymmetry↓, humoral immunity titers↑; White, Red and Green:non-defect chick↑; All light: plasma corticosterone↓, plasma serotonin concentrations↑
Özkan et al., 2022	Brown Nick, H&N International, Oztavuk, Bursa, Turkey	Dark; White, 500K; Green, 8000K	150-250 lux	16L:8D, E0-21		White: gentle pecking at 16 wk↑, severe pecking at 24 and 32 wk↑, the hypothalamic expression of CRH, 5-HTR1A, 5-HTR1B↑, 5-HTT gene expression↑than white but equal to DARK.
Li et al., 2021a	Ross 308	White,4100k; Red, 640 nm; Blue, 460 nm; Dark	200 lux	12L:12D	18L:6D	Red light: average air cell temperature↓, Dark: Male bursa of Fabricius weight↑than blue light
Li et al., 2021b	Ross 308	White,4100k; Red, 640 nm; Blue, 460 nm; Dark	200 lux	12L:12D	18L:6D	White and Blue: body weight↑and feed consumption↑than dark during the first 6 h post-hatch; light group: more stable cloaca temperature at 36 h post-hatch; Red light: total IgG↑at d 14 than others
Kliphuis et al., 2024	Hybrid ISA Brown	Green, 520 nm; Dark	400 lux	12L:12D		Light group: fear response↓

**Note:** 23L:1D: 23 h light and 1 h dark; 20L:4D: 20 h light and 4 h dark; 18L:6D: 18 h light and 6 h dark; 16L:8D: 16 h light and 8 h dark; 12L:12D: 12 h light and 12 h dark. E: embryonic day; E0-21: light exposure from E0 to E21;

All indicators listed in the table exhibit statistically significant differences (*P*<0.05).

### Promotion of feed intake and digestion

Archer et al. (2009) reported that Cobb broiler chicks incubated in darkness exhibited lower feed intake at 42 d of age compared to those exposed to light (12L:12D, 550 lux). Özkan et al., 2012a, Özkan et al., 2012b) demonstrated that at 35 d of age, broilers incubated under continuous light were 94 g and 78 g heavier than those under 16L:8D and dark control groups, respectively. Li et al. (2021) investigated feeding behavior in Ross 308 broilers within 6 h post-hatch and found that chicks incubated under white and blue light consumed significantly more feed than those in the dark group, which may be attributed to light exposure influencing ocular development in chicks (Tang et al., 2023). At the end of incubation, the embryo’s positions within the egg causes greater light to the right eye compared to the left eye (Koshiba et al., 2003). Light-exposed chicks during incubation also exhibit different feeding behaviors: when foraging for grain in pebble-laden environments, those incubated under light were more successful by using their right eye or both eyes, whereas dark-incubated chicks show no eye preference. Thus, light incubation enhances the accuracy of right-eye-mediated grain recognition and pecking, while dark incubation may suppress this ability of chicks (Rogers et al., 2007).

Soliman and El-Sabrout (2020) observed that broilers reared under blue (450 nm) and green light (550 nm) achieved higher body weights and improved feed conversion ratio compared to red light (700 nm). Green light incubation (525 nm) has been shown to enhance intestinal mucosa structure in broiler chicks, improving nutrient digestion and utilization during late incubation (Zhao et al., 2022). These effects may collectively explain the superior growth performance of light-incubated broilers.

### Effects on immune function

Li et al. (2021a) reported higher *bursa of fabricius* weight in broilers incubated in darkness compared to those under blue light (200 lux). Archer et al. (2017) demonstrated that white light and red light (250 lux) during incubation significantly increased IgG antibody titers in 42-d-old broilers compared to that in dark incubation. Li et al. (2021b) observed higher IgG concentrations in 14-d-old chicks incubated under red light than in those under darkness, blue, or white light. Green light promotes the proliferation of thymic T cells (Chen et al., 2016), splenic T lymphocytes (Guo et al., 2017), and bursal B lymphocytes (Li et al., 2015).

Xiong et al. (2020) revealed that green light upregulates melatonin membrane receptor expression in the spleen, accompanied by increased T/B lymphocyte proliferation. In vitro melatonin injection further showed that melatonin enhances green light-induced T lymphocyte proliferation via membrane receptors (*Mel1b, Mel1c*) and nuclear receptors (*RORα/RORγ*), with *Mel1a, Mel1c*, and *RORα* involved in B lymphocyte proliferation. Xiong et al. (2024) proposed a mechanism by which melatonin reduces thymic lymphocyte proliferation: melatonin nuclear receptors *RORα/RORγ* promote pro-apoptotic factors (*Bax, caspase-3*) and pro-inflammatory cytokines (*IFN-γ, TNF-α, IL-6*), while suppressing anti-apoptotic factors (*Bcl-2, Bcl-xL*), suggesting that red light signaling influences thymic lymphocyte apoptosis in broilers. Brennan et al. (2002) found that melatonin administration in *White Leghorn* roosters increased leukocyte, B cell, and T cell counts. These findings suggest that light exposure modulates immune cell function through melatonin secretion.

### Reduction of stress responses

The hypothalamus-pituitary-adrenal (HPA) axis is a key component of the stress response system, and plays a role in early embryonic development (Nordquist et al., 2022). The HPA axis is functional starting from E14 (Jenkins and Porter, 2004). Archer and Mench (2014b) found that chicks incubated under dark conditions exhibited higher fear responses and higher cortisol levels at hatching compared with those incubated under 12 h light and 12 h darkness for 21 days (550 lux). Light incubation enhances post-hatch adaptability and reduces stress responses (Huth et al., 2015; Kliphuis et al., 2024).

Özkan (2012) found that a 16L:8D photoperiod (200-300 lux) during incubation lowered plasma cortisol concentrations in chicks after 8 h of transport. Archer (2013) demonstrated that providing 12 h light cycles (550 lux) during incubation mitigates the impact of stressors on birds. Light incubation reduces stress and fear responses during rearing period, and enhancing poultry welfare (Archer and Mench, 2014a). White, red, and green light (red, 630 nm; green, 520 nm; 250 lux) during incubation significantly decreased plasma cortisol levels and increased serotonin levels in 45-d-old broilers, indicating reduced stress (Archer, 2017). Studies on seagull chicks revealed that light incubation shortened tonic immobility duration, alongside lower plasma cortisol levels (Ruiz-Raya et al., 2022), which means reduced fear responses and stress responses. Monochromatic green light (150-392 lux) significantly reduced vocalization frequency in "Lonely Test" and delayed emergence trials (Yu, 2016), also indicating reduced fear responses. Light incubation also improves cognitive and learning abilities in chicks, enabling better environmental discrimination (Chiandetti et al., 2009) and enhancing adaptation to post-hatch environments (Özkan et al., 2012a).

### Effects on feather pecking

Feather pecking is an important welfare concern in layer hen production. Riedstra and Groothuis (2004) reported that exposing layer eggs to high-intensity incandescent light (750-1000 lux) for 2 h from E18 to E19 increased the frequency of gentle pecking behaviors in adult hens. Light exposure may lower feather pecking by enhancing birds’ ability to discriminate food, further enhancing the ability of foraging (de Haas et al., 2021). Although light incubation has no immediate effect on early pecking behavior in chicks, Özkan et al. (2012b) observed that green light-incubated hens exhibited gradually increasing feather pecking at 16 wks, whereas white light-incubated layers showed higher levels of aggressive pecking at 32 wks. Usually low serotonin (5-hydroxytryptamine, 5-HT) levels exacerbate feather pecking (Van Hierden et al., 2004). Red light increased 5-HT levels and reduced cortisol concentrations in layer breeders, correlating with lower frequencies of severe feather pecking (Shi et al., 2019), while green light during incubation elevates 5-HT levels, promotes non-endogenous circadian rhythms, and accelerates light adaptation, thereby reducing the incidence of feather pecking (Wang, 2021).

## Related mechanisms of light incubation

**Fig. 1:**
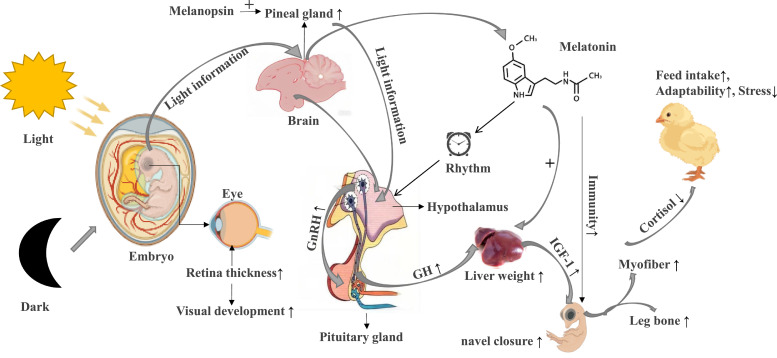
Mechanisms of light incubation.

The related mechanisms of light incubation were summarized as follows (see Fig. 1). (1) Promote the development of the retina and pineal gland, increase melanopin expression and melatonin secretion, directly or indirectly regulating the endocrine activities and behaviors; (2) Promote the development of the somatotropic axis, increasing the secretion of GH and IGF-1, affect the myofiber development; (3) Facilitates the early establishment of circadian rhythms, enhancing post-hatch light adaptability; 4) Affects the corticosterone level, and reduces stress responses in post-hatch chicks.

### The role of photoreceptors

In mammals, light information reaches the retina through the eye and is transmitted to the visual center through efferent neurons in the retina to obtain information relevant to survival. Birds exhibit more sophisticated mechanisms for processing light information. Beyond the retinal pathway, light can penetrate the skull to reach the pineal gland and hypothalamus, where it binds to extraretinal photoreceptors. This interaction regulates endocrine activity and thus physiological functions in birds (Zawilska et al., 1998). As the mediators for photoelectric signal conversion, opsins are categorized into cone opsins and rod opsins. Rod opsins exclusively detect light intensity, while cone opsins perceive color through specific spectral ranges. Researchers isolated a blue-sensitive opsin (pinopsin) from the chicken pineal gland, followed by discoveries of additional opsins including melanopsin, peropsin, neuropsin, and encephalopsin (Tomonari et al., 2007). Poultry can perceive light stimulation during the incubation at E8, chicken pineal cells begin to differentiate into melatonin-producing cells and initiate melatonin secretion (Grechez-Cassiau et al., 1995). Red- and green-sensitive opsins appear from E14, and pineal opsins demonstrate light sensitivity from E16 (Lamosová et al., 1997).

### The role of melanopsin

Melanopsin, a non-visual photopigment, was first isolated from the skin melanocytes and retina of Xenopus laevis. Recent studies have demonstrated its expression in the retina, pineal gland, and suprachiasmatic nucleus (SCN) of poultry (Chaurasia et al., 2005; Nayak et al., 2007), with particularly high levels observed in the pineal gland (Holthues et al., 2005). In mammals, light signals are transmitted via melanopsin-expressing retinal ganglion cells through the retinohypothalamic tract to the SCN. The signal is subsequently relayed to the paraventricular nucleus, then to the intermediolateral cell column of the spinal cord, and finally to the superior cervical ganglion and postganglionic neurons of the pineal gland to regulate melatonin synthesis. Melatonin conveys photoperiodic information throughout the body, directly or indirectly modulating endocrine functions, behavior, and other physiological processes in animals (Walton et al., 2011).

Animals rely on light to form vision, enabling them to perceive the shape, color, and movement of objects. Variations in light intensity and periodicity also contribute to non-image-forming vision, which facilitates pupillary reflexes, circadian rhythms, and light-dark perception, thereby regulating animal behavior and physiology. In the photodetection process, beyond the roles of retinal rod and cone cells, an independent regulatory pathway exists that depends on melanopsin secreted by retinal ganglion cells. This pathway retains photoreceptor functions even in mice with defective rods and cones. However, knockout of the melanopsin gene results in a complete loss of light detection, highlighting the melanopsin's critical role in light perception (Lucas et al., 2003). Compared to the 16L:8D photoperiod, the intermittent lighting group (12L:2D:4L:6D) exhibited significantly upregulated Opn4 mRNA expression in the pineal gland, while the 12L:12D group showed downregulation in laying hens, indicating a relationship between melanopsin expression and photoperiod (Geng et al., 2022).

Fahrenkrug et al. (2004) detected the expression of melanopsin gene -Opn4 mRNA in rats as early as E18, but light-responsive function only emerged postnatally. Circadian rhythmicity in Opn4 mRNA expression has been observed in the rat retina (Hannibal and Fahrenkrug, 2004), and similar rhythmic patterns occur in the pineal gland and retina of chickens (Holthues et al., 2005). In mice, retinal Opn4 mRNA levels under continuous light (24L) were significantly lower than under a 12L:12D photoperiod (Wu et al., 2012). Conversely, the pineal gland can directly perceive light and regulate circadian clocks and melatonin secretion via melanopsin (Natesan et al., 2002). Opn4 mRNA content in the chicken retina under 6L:18D was threefold higher compared to longer photoperiods (Lima et al., 2006).

### The role of melatonin

Melatonin has a wide range of effects on poultry. It enhances the activity of immune organs and immune cytokines, thereby boosting the overall immunity (Xiong et al., 2024), and exhibits anti-inflammatory properties (Lu et al., 2019). Avian reproductive activities are also regulated by melatonin; reducing photoperiods or administering exogenous melatonin suppresses gonadal activity and delays sexual maturation (Lewis et al., 2006). As a natural anti-stress agent, melatonin mitigates production-related stress via the hypothalamic-pituitary-adrenal (HPA) axis (Zhou et al., 2024). Additionally, melatonin can alleviate oxidative stress, scavenges free radicals (Zhang et al., 2021), improves sleep quality, and regulates circadian rhythms (Drozdova et al., 2019).

Melatonin is mainly secreted by the pineal gland, with its content increasing at night and decreasing during the day (Feng, 2017; Matsuo et al., 2013; Wang, 2021). A significant negative correlation between pineal melanopsin mRNA expression and melatonin secretion in laying hens were observed (Geng et al., 2022).

Light incubation regulates the development and maturation of the pineal gland, thereby influencing melatonin secretion (Tang et al., 2023). Furthermore, melatonin enhances embryonic growth by elevating hepatic IGF-1 secretion (Wang et al., 2014; Wang, 2021). Under monochromatic green light, melatonin reduces somatostatin (SST) secretion via the melatonin 1b (Mel1b) and melatonin 1c (Mel1c) receptors (Qin et al., 2021; Yue et al., 2019).

Drozdova et al. (2019) found that the light-dark cycle (12L:12D) promotes rhythmic melatonin secretion in pineal gland of chicken embryo, with varying melatonin levels under different light spectra (white, red, green, and blue light). The red light group exhibited the highest melatonin secretion, while the blue light group showed the lowest. Notably, the largest amplitude of melatonin secretion was observed under red and blue light, potentially indicating circadian rhythm entrainment. Wang (2021) demonstrated that green light incubation enables the establishment of circadian rhythm before E17 in White Leghorn, Barred Plymouth Rock, Columbian Plymouth Rock, and Rhode Island Red embryos. Green light enhances the expression of positive regulators in the cellular clock mechanism (*cCLOCK, cBMAL1, cBMAL2, and cAANAT*), thereby stimulating melatonin secretion in broilers (Cao et al., 2017).

Bian et al. (2019) observed that exposing chicks to monochromatic light (white, red, green, and blue) induced significant circadian rhythms in melanopsin genes (*cOpn4-1, cOpn4-2*), circadian clock genes (*cBmal1, cCryl, cPer2, cPer3*), the melatonin rate-limiting enzyme gene (cAanat), and melatonin concentration, and no such rhythms were established under constant darkness. Green light promotes the expression of circadian clock genes (*Clock, Bmal1, Bmal2*) in the pineal gland (Jiang et al., 2016) and hypothalamus (Jiang et al., 2017), thereby enhancing melatonin secretion. Tang et al. (2023) found no rhythmic melatonin secretion under different light spectra but noted light-specific effects on secretion levels, with the highest plasma melatonin concentrations under green and blue light and lower under darkness and white light.

### Early formation of circadian rhythm

Circadian rhythms in poultry begin to form during incubation. Unlike mammalian embryos, chicken embryos cannot directly acquire hormonal signals from the maternal bloodstream to establish circadian rhythms and instead rely on external zeitgebers, with the most critical being regular light-dark cycles (Bell-Pedersen et al., 2005). The circadian rhythms established during embryogenesis influence both the timing of hatching and post-hatch circadian patterns in chicks for at least three days (Torii et al., 2007). Özkan et al. (2012b) investigated melatonin rhythms in broiler embryos under three light regimes: 16L:8D throughout incubation, 16L:8D during the final week, and continuous 24 h darkness. They revealed that the 16L:8D throughout incubation exhibited diurnal melatonin rhythms at E19 and post-hatch, whereas the continuous darkness group showed no rhythms. The final-week 16L:8D group displayed rhythms only at post-hatch, indicating that the 16L:8D throughout incubation promoted circadian entrainment by 1-2 days compared to other groups. Drozdova et al. (2019) further demonstrated that 12L:12D exposure to different light wavelengths during incubation induced melatonin rhythmicity as early as E20, highlighting the significant influence of light on early rhythm formation.

Pilorz et al. (2016) confirmed that melanopsin directly mediates light-induced corticosterone secretion, with corticosterone levels significantly increasing under light exposure. Notably, light-induced corticosterone secretion in embryos is inversely correlated with melatonin secretion (Cutolo et al., 2006; Tong et al., 2018). Light incubation induces significant changes in plasma melatonin and corticosterone levels in chicks (Özkan et al., 2012a) and elevates growth hormone levels in hatchlings (Bai et al., 2016).

## Summary and future perspectives

During the incubation, the light perception function of poultry embryos has already developed. Light incubation can influence embryo development, hatching performance, and post-hatch performance. Key components of poultry’s light-sensing system, including the eyes, retina, and the pineal gland are mature during incubation. Periodic light incubation can help establish early circadian rhythms, enhance post-hatch adaptability, and reduce stress responses. Light stimulation also activates the embryonic growth axis, promoting the secretion of hormones such as GHRH, GH, and IGF-1. These hormones facilitate navel closure, and the development of leg bones, myofibers, ultimately improving chick quality and neonatal health at hatching. Light incubation affects visual development in embryos, influencing post-hatch feeding behavior and performance.

Light incubation has obvious impacts on embryonic development and post-hatch performance of poultry, with variations in different experimental conditions, such as light spectrum, intensity, photoperiod, light exposure age and duration, poultry breed, egg characteristics, etc., resulting in inconsistent and diverse outcomes. Especially for light spectrum and intensity, most previous studies measured the intensity of varying color lights by using “lux” unit, which is based on human visual response. This means that for chickens, the intensity of colored light such as blue and green is much brighter than that of white light, resulting in an interaction effect between wavelength and light intensity. However, for the sake of comparison, the lux was not converted to “clux” in this article. Further systematic research is needed to elucidate the detailed mechanism of light incubation and to optimize more parameters, such as maternal traits, egg characteristics, detailed light intensity, spectrum, photoperiod, light exposure duration, etc. for commercial incubation of chickens. This will lay a scientific foundation for fully utilization of light to improve the performance and welfare of poultry.

## CRediT authorship contribution statement

**S.Z. Yu:** Data curation, Investigation, Methodology, Writing – original draft, Writing – review & editing. **L.F. Cheng:** Data curation, Investigation. **M.Y. He:** Data curation, Investigation. **J. Cao:** Data curation, Investigation. **A.L. Geng:** Conceptualization, Data curation, Funding acquisition, Investigation, Methodology, Writing – original draft, Writing – review & editing.

## Disclosures

The authors declared that we have no conflicts of interest to this work.
